# Diseases of the Digestive Organs

**Published:** 1847-06

**Authors:** N. S. Davis

**Affiliations:** Binghamton


*A Few Observations on some of the most common Diseases of the Digestive Organs: By* N. S. Davis, M. D., *Binghamton*, 1847.—Perhaps there is no class of diseases more frequently neglected or badly managed, than those which occur most frequently, and at the same time are not attended with any immediate danger to life. Of this class, are those chronic and annoying affections usually included under the head of Dyspepsia. And their very frequency is the cause of their being so often misunderstood, or carelessly treated. For it is a general rule, that whatever is most common, is least apt to excite in the mind either careful attention or close thought.

Chronic diseases of the digestive organs may be divided into two great classes. The symptoms presented by both are exceedingly various. They may be described in general terms, however, as follows:—The first, in its early stage, is characterized by a simple loss of action in the muscular coat of the stomach and intestines, causing the bowels to be costive; the head often dizzy or painful; not unfrequently paroxysms of what is popularly termed sick-headache; simple irritability of the nervous system; a general sense of lassitude or indisposition to active exertion, either mental or physical; and a speedy sense of weariness on attempting such exertion. The patient often says he does not feel well, or able to labor, but he can scarcely tell why; for in the early stages the appetite often remains good, sometimes morbidly so; the tongue moist, and only slightly furred; and the pulse natural. But, at a later period, indigestion is more prominent, giving rise to more or less distress of some kind in the epigastric region, soon after eating. Not unfrequently, however, (particularly in females,) the appetite is entirely lost.

The second class of patients are almost uniformly characterized by a clean red tongue, a quicker pulse, more acidity in the stomach, more painful digestion, particularly more sense of heat or burning in the stomach, more loss of flesh, and the costiveness often alternating with diarrhea. And here again the food is often habitually rejected almost as soon as it is swallowed.

The two classes of symptoms here described, exist in every grade of severity, and often run into each other, making a case of no little complexity. And not unfrequently, the first, after a protracted existence, or bad management, terminates in the second.

Let us now inquire what is the real *pathology* of these diseases.

There is no proposition more susceptible of proof, than that the whole nervous system, (and no part of it more so than that termed organic,) is dependent on due supply of oxygen in the arterial blood, for the healthy performance of its functions. Another universally admitted proposition is, that all muscular contraction is dependent on nervous stimulation or agency; and not only so, but all the capillary vesssels; and consequently the secretions are more or less under the influence of the same agency. And still another well established proposition is, that in the same proportion that the tone, or healthy energy of the nervous system is impaired, its irritability or susceptibility to impressions is increased. Keeping these general propositions in view, let us return to the diseases in question. They are well known to occur most frequently in the female sex, and especially among the higher or more fashionable circles; next among men of sedentary habits, hard students, and such as have impaired the vigor of their organic systems by practising some of the vices of the age; and none more frequently than excessive venereal indulgence and onanism.

Now, why do dyspeptic complaints arise principally among these classes? Simply because the first, by the foolish fashion of confining the chest, and the second, by the want of exercise, fail to receive the necessary quantity of oxygen into the lungs; consequently, the nervous system is not duly supported by well oxygenated arterial blood; and hence its influence over the whole muscular system is impaired; and hence too the sense of lassitude or indisposition to active exertion in the cerebral or voluntary systems, and the loss of peristaltic motion, and consequent costiveness, occasioned by the diminished energy of the organic system.

But this is not all. The same state of the nervous system causes a diminution of all the secretions. The gastric juice is diminished in quantity, and the food is consequently slower and more difficult of digestion. Many of the excretions are also partially retained, vitiating the blood, and giving rise to the frequent attacks of sick-headache, and a great variety of other distressing symptoms. The third and fourth classes of men produce the same results, but in another way. The third err by concentrating the vital energy too much and too long on the cerebrum alone, thereby impairing the organic nervous system, and producing all the subsequent morbid phenomena already named; while the fourth and last produce the same consequences by a direct and excessive drain on the organic system itself.

I might here detail a number of well marked cases of this last class; but the practice of every close observer will furnish him abundance of illustrations, and therefore it is unnecessary. It will be observed that all these causes produce only the first class of causes described above. And hence the inquiry will arise, from whence comes the second class? Every intelligent physician will quickly recognize in the second, simply a more or less severe inflammation of the inner coats of the stomach; and consequently it may arise from any of the ordinary causes of inflammation. But I venture the assertion that nine cases out of every ten arise from either neglect or mal-treatment of the first class. Nothing is more common than to find individuals, when they first begin to suffer some of the earliest symptoms I have named, resorting to some of the thousand kinds of patent pills with which the country is filled; and dose after dose is taken, each, by temporarily unlocking the secretions, affording an evanescent and deceptive relief—just sufficient to keep up the patient's perseverance in their use—until (I speak within bounds when I say it,) *tons* of pills are annually consumed in our country, for this very purpose. Not only so, but scores of physicians, and some too of high standing, do the same thing, with different means. Let a patient of this kind call on one of these physicians, and they are promptly told that they are *bilious*, (a term used to cover more ignorance than any other in the whole medical nomenclature,) and given an emetic, a dose of calomel, or a box of blue pills, and sent home. The patient takes it, experiences the same temporary relief as in the other case; praises the doctor's prescription, and in a week or fortnight returns for another.

Now the difficulty with this treatment is, that the remedies have not the remotest influence over either the remote or proximate causes of the morbid condition of the patient. On the contrary, the temporary stimulus of the remedy on the nervous fibrils distributed on the inner coats of the stomach and intestines, causes a temporary flow of the secretions, and consequently a short season of relief. But every repetition of this direct stimulation of these nervous fibrils, increases their irritability, until in due course of time we get the smooth red tongue, the frequent vomiting, and occasional diarrhea: in a word, all the symptoms of confirmed chronic inflammation of the villous or mucus coat of the digestive canal, or the worst form of dyspepsia. The patient now soon gets tired of his *regular* doctor, and commences the interminable round of quackery.

I had here intended to illustrate the foregoing views, by a detail of several cases from my note-book; but the lateness of the hour when I commenced this communication rendered it impracticable. And it is the less to be regretted, as such cases are within the observation of us all. Neither is it my intention to go into any details of treatment; for if our pathology is correct, the appropriate treatment will readily suggest itself to every intelligent mind. Obviously the first thing to be done is, to remove the remote and exciting causes. This is a *sine qua non*, without which no *permanent* relief can be secured. The patient must have a sufficient quantity of pure air, and well oxygenated blood; due physical exercise; a plain, simple diet; mental relaxation; and a removal of all undue drains on the organic system of nerves, &c.

The direct therapeutic agents employed must be so combined, that while they soothe the irritability and sustain the strength of the whole nervous system, they shall gently promote all the secretions and procure a regular evacuation of the bowels once a day; carefully avoiding all harsh remedies of every description. This is easily done by properly combining gentle tonics and anodynes with just sufficient laxative medicine to procure the regular evacuation of the bowels; and occasionally the addition of a mercurial alterative will be beneficial.

I make these suggestions for two purposes: first, to contribute my mite to the doings of the Society; and secondly, to call up more serious reflections on these common diseases.—*Transactions of N. Y. Med. Society.*

